# Case Report: Giant paratesticular liposarcoma was resected and refused radical orchiectomy

**DOI:** 10.3389/fonc.2023.1223081

**Published:** 2023-08-11

**Authors:** Qianming Zou, Shu Gan, Yuan Li, Qinzhan Huang, Shusheng Wang, Siyi Li, Chiming Gu

**Affiliations:** Department of Urology, The Second Affiliated Hospital of Guangzhou University of Chinese Medicine, Guangzhou, Guangdong, China

**Keywords:** paratesticular liposarcoma, scrotal mass, early diagnosis, radical orchiectomy, pathology

## Abstract

Paratesticular liposarcoma (PLS) causes scrotal mass changes, rarely in the urinary system. Before surgery, PLS causes scrotal mass changes that are difficult to distinguish from other causes. There has been a report of a giant paratestis liposarcoma resection and refusal to undergo orchiectomy. A 65-year-old man presented with finding the left scrotal mass after 2 years. Physical examination showed that the left scrotal mass was obviously difficult to retract. Pelvic CT showed that the left scrotal mass and flaky fat density shadow accompanied with left inguinal hernia. During surgery, laparoscopic exploration was performed to rule out inguinal hernia, and a scrotal exploration was also performed concurrently. The intraoperative frozen pathology considered lipogenic tumor, and the patient’s wife refused to undergo simultaneous left radical orchiectomy. Later the mass was completely removed, and postoperative pathology confirmed paratestis liposarcoma. During a 15-month routine follow-up, the tumor did not recur locally or metastasize distantly. PLS should be focused on early diagnosis and treatment, preoperative examinations and postoperative pathology should be combined, and highly personalized treatment will be implemented.

## Introduction

Liposarcoma (LPS) is a malignant tumor of adipocyte differentiation, approximately 20% of all soft tissue sarcomas are liposarcomas (1). Liposarcomas are mostly found in the deeper tissues of the retroperitoneum and extremities. They are rare in the paratesticular area. Most liposarcomas are well-differentiated and dedifferentiated (WDLPS/DDLPS) (2).

Approximately 7% of all scrotal tumors are paratesticular liposarcomas (PLS).

Only about 272 cases have been reported worldwide (2–7). PLS are tumors that originate in the scrotum rather than the testis, in addition to the epididymis, the spermatic cord, and the tunica vaginalis may also be involved. PLS is often misdiagnosed before surgery as a painless mass of the scrotum. Early and prompt surgical treatment can provide the best outcome. If it is suspected to be paratesticular liposarcoma, any suspected fatty swelling should be thoroughly investigated and promptly removed, as delayed treatment can lead to poor outcomes.

We report a case of massive paratestis liposarcoma that was resected and refused to undergo radical testicular resection. By reviewing the diagnosis and treatment process of PLS and combining it with relevant literature reports, which provides a reference for diagnosing and treating the disease.

## Case description

**Figure 1:**
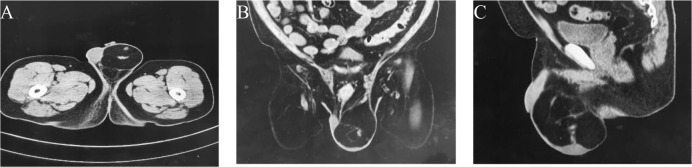
The enhanced CT scan reveals patchy fat density shadows on the left scrotum. **(A)** Axial view. **(B)** Front view. **(C)** Side view.

A 65-year-old man presented to our hospital on December 21, 2021, with “finding a left inguinal mass over 2 years”. Diabetes, coronary heart disease, smoking history, and no alcohol consumption were present in the patient. Previous removal of the right testicular cyst in 2018 showed no malignant changes. He denied having any relevant tumour history or family history. On examination, there were enlarged left scrotum, without erythema or pain, with indistinct borders, approximately 15 cm x 8 cm, with the mass extending into the left inguinal region. Inability to palpate the left testicle and epididymis. The trans illumination test was negative and the left scrotal mass was obviously difficult to retract. Laboratory examinations showed that HCG: 0 IU/L, AFP: 1.91ng/ml. On ultrasound, the left inguinal canal showed a mass, which was considered an inguinal hernia (content considered to be mesenteric tissue, with a high probability of incarceration). Pelvic CT plain scan and enhancement showed a left inguinal hernia, with content being a small amount of mesenteric fat tissue in the left inguinal region., a mass-like fatty density lesion was seen in the left testicle, which was considered to be a vascular muscle adipose tumor or a fatty tumor (**Figures 1A–C**). The patient was tentatively diagnosed with a left inguinal hernia with a left scrotal mass based on his associated symptoms and examination.

**Figure 2:**
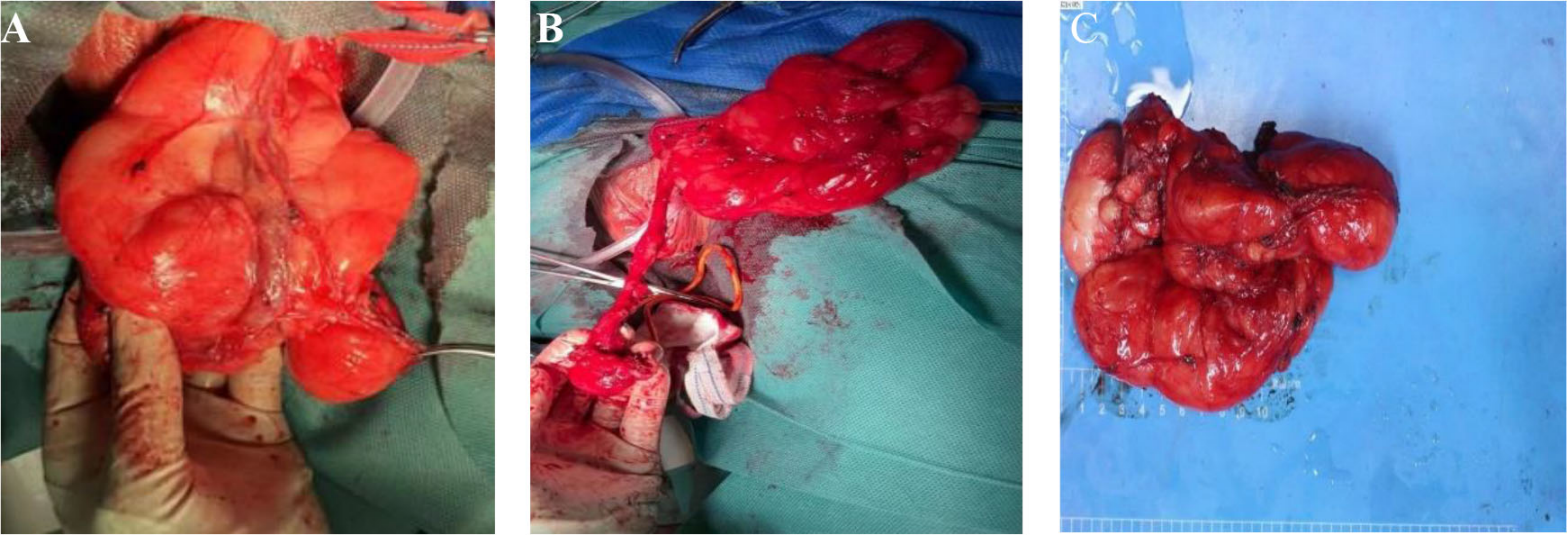
**(A)** A fatty tumour in the left inguinal region that surrounded the testicular vein and spermatic cord. **(B)** Preserve the spermatic cord and testicles. **(C)** The tumor envelope is intact, with some areas nodular, greyish-yellow in texture and soft.

After evaluating the patient’s condition and communicating with them, a laparotomy laparoscopy exploration was performed and a testicular exploration was also conducted. During the surgery, the laparoscopic exploration excluded an inguinal hernia, while the testicular exploration revealed a fatty tumour in the left inguinal region that surrounded the testicular vein and spermatic cord (**Figure 2A**). A frozen pathological examination during the operation suggested a fatty tumour-like tumour. After explaining the situation and pathological findings to the patient’s wife, she refused to perform a concurrent radical resection of the left testicle. The tumour was then resected completely (**Figure 2B**). The postoperative pathological examination confirmed a high-grade liposarcoma adjacent to the spermatic cord. The patient recovered well after the surgery, and no adjuvant treatment was given. A pelvic MR scan was performed every 6 months, the tumor did not recur locally or metastasize distantly during a 15-month follow-up.

**Figure 3:**
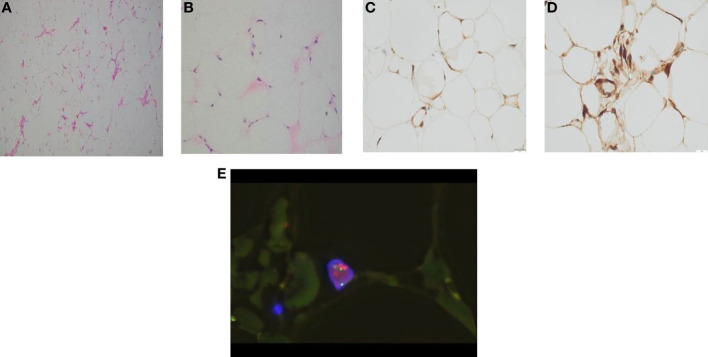
**(A)** HEx200, the tumor is composed of mature fat, with thin-walled capillaries visible in the stroma. **(B)** HEx400, high magnification display of enlarged nuclei and atypical adipocytes. **(C)** S-100 immunohistochemistry shows positive tumor nuclei and cytoplasm. **(D)** P16 immunohistochemistry showed positive cytoplasm of tumor cells. **(E)** WDLPS MDM2 FISH: MDM2/CSP12: 9.10, MDM2 mean copy number: 18.20.

The gross pathological examination showed a 18 × 8 × 5 cm tumour (**Figure 2C**), the tumor envelope is intact, with some areas nodular, greyish-yellow in texture and soft. The pathological examination showed well-differentiated adipocytes in most of the tumour, with a few cells showing slightly abnormal nuclei (**Figures 3A, B**). The genetic testing results were consistent with an atypical lipid tumor/well-differentiated liposarcoma. MDM2 gene amplification was detected in the molecular pathological examination (**Figure 3E**). Immunohistochemical findings: S-100 (+) (**Figure 3C**), P16 (marked 2 places, all+) (**Figure 3D**), P53 (-), Ki67 (<1%), CD34 (partially+). The amplification of the MDM2 gene supported the diagnosis of WDLPS.

## Discussion

Paratesticular liposarcoma is a rare tumour, and most paratesticular liposarcomas typically affect adults between the ages of 50 and 60 (8, 9). Existing lipomas may be converted to PLS by malignant transformation or by multiplication from the peripheral fat tissue of spermatic cord (6). According to the 2020 WHO Soft Tissue Classification, liposarcomas have various pathological types, such as well-differentiated liposarcomas, dedifferentiated liposarcomas, myxoid liposarcomas, pleomorphic liposarcomas, and myxoid pleomorphic liposarcomas. Different types of molecular basis, clinicopathological features and biological behaviours differ (10). There are a small number of liposarcomas that are mixed types, which have features of at least two subtypes combined (11). Over half of PLPS patients have WDLPS with a favorable prognosis. WDLPS can recur with low metastatic rates (12). The main route of metastasis for this tumour is through the inguinal canal that extends to the peritoneal cavity, and rarely through the blood and lymphatic pathways. If WDLPS is not diagnosed in time, it may transform malignantly into DDLPS with a worse prognosis (5). These tumors present as slowly growing, painless inguinal swellings that are usually misdiagnosed as inguinal hernias, hydrocele, testicular tumors, epididymitis, orchitis, tuberculosis, and lipoma (13, 14). Physical examination and ultrasound cannot distinguish these entities from lipomas, especially in small which may be misinterpreted as benign features (15, 16). Considering the size and degree of adhesion, identifying its origin can be difficult (17).

Here we reported a male with a giant PLS that had molecular changes typical of WDL. Tumours >10cm in diameter are defined as “giant”, there are only a limited number of these cases which reported in the English literature until now (**Table 1**)

**Table 1:** Case reports of primary giant PLS.

Author	Age	Type	Duration of growth	Side	Size (cm)	Pain	Original diagnosis	Primary Surgery	Margin	CT Scan	Postoperative-Treatment
Pavone, G(2022) (7)	76	WDL	2y	Right	14.5	No	inguinal hernia	tumor resection	R0	Negative	rescue orchiectomy
Chan, K. (2022) (4)	87	WDL	11m	Left	17×15	No	inguinal hernia	Orchiectomy and high spermatic cord ligation	R1	metastatic lung deposits	None
Li, J(2022) (6)	53	DDL	3y	Left	15×9	No	Suspicious scrotal mass	tumor resection and orchiectomy	R0	Negative	None
Noguchi, T (2020) (12)	75	DDL	1y	Right	26	No	Suspicious scrotal mass	radical orchiectomy and lymph node dissection	R0	multiple lung nodules	gemcitabine plus docetaxel chemotherapy
Keenan (2019) (16)	82	DDL	1.5m	Left	11×9	Yes	Scrotal hematoma	Hemiscrotectomy	R1	Positive (Pelvis)	Palliative RT
Mouden, K (2019) (18)	55	WDL	2y	Left	15×17	Yes	Suspicious scrotal mass	orchiectomy and hemiscrotectomy and pelvic tumor resection	R1	Positive (Pelvis)	None
ZUWEI LI (2018) (15)	51	DDL	2m	Right	13×8	No	spermatocytoma	tumor resection and orchiectomy	R0	Negative	None
Sopena-Sutil (2016) (19)	56	WDL	25y	Left	40×40	No	Liposarcoma	Orchiectomy and spermatic cord ligation	R0	Negative	None
Grossi (2014) (11)	81	WDL/MRCL	4y	Right	28×30	No	Suspicious scrotal mass	Orchiectomy and high spermatic cord ligation	NA	Negative	None

MRCL, myxoid/round cell liposarcoma; RT, radiotherapy; NA, not available; R0, no residual disease after primary surgery; R1, residual disease after primary surgery.

PLS can be difficult to diagnose preoperatively, which can affect surgical treatment outcomes. Malignant tumors exhibit rapid growth, large volume and pain. In cases of suspicion, preoperative CT and/or MRI could be useful to offer the relevant information (20). Contrast agent computed tomography is the most commonly used maging method for diagnosing possible soft tissue sarcomas of the pelvis. Nodular septa within soft tissue mild to moderately enhanced attenuation helps distinguish it from benign lipomas, In addition, CT helps determine the location, stage and follow-up after treatment (21). MRI remains to be the standard of gold for the examination of soft tissue tumours because of its higher soft tissue resolution, which helps to characterise and depict the extent of local tumour extension and hence staging (4, 16). In our case, preoperative MRI was not investigated because no PLS had been suspected, postoperative follow-up using MRI to assess the presence of local recurrence.

Postoperative histopathology, immunohistochemistry and cytomorphological features are still the gold standard for determining PLS. WDLPS and DDLPS are related in cytogenetics and share basic genetic abnormalities in sequences amplified from the chromosome 12q long arm (22), which carries the oncogenes MDM2, CDK4 and HMGA2, the co-amplification of MDM2 and CDK4 is a meaningful marker for the confirmation of the diagnosis of WDLPS/DDLPS, it is important to confirm the diagnosis of WDLPS/DDLPS by co-amplifying MDM2 and CDK4, while HMGA2 rearrangements can occur in benign lesions as well as other types of cancer (23, 24). MDM2 is a major target of tumor therapy, and wild-type activities of p53 can be restored by blocking interference with MDM2 and p53. In our case, immunohistochemistry findings and MDM2 gene amplification confirmed the diagnosis of WDLPS.

Extensive resection locally coupled with radical orchiectomy and high ligature of the spermatic cord in the early stage is the standard treatment for liposarcoma. It is not recommended to dissect retroperitoneal lymph nodes unless they have metastasized (15). As the clinical presentation of PLS resembles that of a scrotal lipoma or scrotal tumour or inguinal hernia, when suspected PLS is diagnosed, immediate radical surgery to prevent local recurrence and poor prognosis should be performed. In a study that included 265 cases of PLS, patients undergoing high inguinal orchiectomy have significantly higher relapse free survival rates than those undergoing simple tumor resection, patients with positive edges are more likely to recur than those with negative edges (3). Incomplete excision is associated with frequent recurrence (18). To reduce the possibility of local relapse, it remains controversial whether resections should be extended to include unaffected organs adjoining the original tumour.

Two retrospective analyses of retroperitoneal WDLPS showed no association between survival benefits associated with organ resection and RP WDLPS (25) and the incidence of organ infiltration was low in recurrent RP WDLPS patients (26), the data suggest that OS and DFS were associated with organ removal, whereas postoperative complications were less likely to occur with organ preservation, therefore, it is important to consider the preservation of organs unaffected by the disease. So we believe in PLS, when intraoperative rapid freezing confirms the pathology, extensive local excision should be performed, and if necessary, radical testicular surgery should be performed simultaneously. Selective resection of adjoining organs only if invasion is suspected clinically (6).

In our case, the intraoperative frozen pathology considered lipogenic tumor, the surgical methods, including radical orchiectomy, extensive local resection, and high ligation of the spermatic cord, which had been recommended, but the patient’s wife refused to undergo simultaneous left testis radical resection, so we had to choose to extend the local excision. Postoperatively, the patient was suggested to perform an additional high inguinal orchiectomy, but the patient refused, agreeing to regular postoperative follow-up for early detection of tumor recurrence and distant metastases. Recurrent disease can be treated with surgical re-excision when possible.

Moreover, the literature on the benefits of adjuvant therapy after complete surgery remains controversial (19), as the number is small. Because of its rarity, the adjuvant treatment of this malignancy has no consensus. Negative cut margins in radical surgery are the most important factor in reducing local recurrences, the 3-year relapse-free local survival rate was reported to be 100% on negative margins and 29% on positive margins (15). By changing the existing concepts associated with retroperitoneal sarcoma, consideration should be given to adjacent radiotherapy in the case of positive margins and chemotherapy in the case of distant metastatic risk (large tumour, high grade and proliferation index, necrosis) (27). Nevertheless, whether radiation therapy should be used as routine postoperative treatment is still open to debate, as tumours that recur after radiotherapy can be more invasive (28). In the study that included 265 cases of PLS (3), the results showed that adjuvant radiotherapy after surgical therapy had no significantly impact on recurrence free survival, even in the subgroup of patients with positive cut margins analyzed. Large-scale research did not support the benefit of adjuvant radiotherapy in reducing local recurrence. There are no large-scale studies on chemotherapy outcomes. For liposarcoma with distant metastases, a DXR-containing first-line chemotherapy regimen is recommended, the effective rate of first-line chemotherapy for dedifferentiated liposarcoma is only 25% (doxorubicin 8%, doxorubicin plus cyclophosphamide 17%) (29). Eribulin, an inhibitor of the growth of microtubules, is very effective in patients with advanced liposarcoma as a second-line therapy (30). In a case with massive parasternal liposarcoma that had metastasized to the lungs, gemcitabine combined with docetaxel therapy was reported to have controlled the cancer after one year (12). There is limited evidence for the effectiveness of chemotherapy for PLS, but we believe that chemotherapy would help improve survival in patients with advanced or distant metastases, in some studies, chemotherapy was suggested as a treatment for high grade LPS.

Additionally, further study on molecular characterization will help to develop new therapeutic approaches targeting drugs or understanding the causes and risk factors in patients with PLS. CDK4 amplification or MDM2-p53 pathway coactivator inhibitors might represent new treatment targets for WDLPS/DDLPS. There are currently several drugs being developed that target the MDM2 gene, including Palbociclib (31). The correlation of CDK4/6 with the MDM2 inhibitor (HDM201) or the mTOR inhibitor (everolimus) seems promising in particular (32).

To date, research on the prognosis of PLS have been rather limited, prognosis and overall survival vary according to a number of risk factors, including tumour grade, size (tumours larger than 5cm), depth of infiltration and histopathology classification (15). As the prognosis may depend on surgical techniques and the presence of distant metastasis, it is necessary to improve surgical techniques to reduce the occurrence of positive surgical margins, and closely follow up after surgery to detect the possibility of distant metastasis in the early stage, in order to improve the patient’s prognosis. To control local recurrence, extensive local resections often need to be repeated.

In conclusion, rarely diagnosed giant PLS results in delayed treatment due to misdiagnosis, there is no consensus on its management currently. The diagnosis may be challenging but a thorough history, exam and image (US, CT and MRI) with histopathological findings will provide a clear diagnosis. Radical orchiectomy with extensive local excision and high ligature of the sperm cord provides the best results when a preoperative diagnosis or high suspicion is present. The value of adjuvant treatment with radiotherapy and chemotherapy has yet to be determined because there have been no large-scale PLS clinical trials conducted yet. Long-term monitoring is required as local recurrence and distant metastases are possible.

## Data availability statement

The original data that supports the conclusions of the article will be provided by the authors without any improper reservation.

## Ethics statement

Written informed consent was obtained from the individual(s) for the publication of any potentially identifiable images or data included in this article. Written informed consent was obtained from the participant/patient(s) for the publication of this case report.

## Author contributions

QZ compose the manuscripts. SG provide literature review. SW and YL study supervision. QH and SL formal analysis. CG review and editing. All authors contributed to the article and approved the submitted version.
